# Medicaid Expansion Produces Long-Term Impact on Insurance Coverage Rates in Community Health Centers

**DOI:** 10.1177/2150131917709403

**Published:** 2017-05-17

**Authors:** Nathalie Huguet, Megan J. Hoopes, Heather Angier, Miguel Marino, Heather Holderness, Jennifer E. DeVoe

**Affiliations:** 1 Oregon Health & Science University, Portland, OR, USA; 2 OCHIN Inc, Portland, OR, USA

**Keywords:** health policy, community health centers, Affordable Care Act, United States

## Abstract

**Background:**It is crucial to understand the impact of the Affordable Care Act (ACA). This study assesses changes in insurance status of patients visiting community health centers (CHCs) comparing states that expanded Medicaid to those that did not. **Methods:** Electronic health record data on 875,571 patients aged 19 to 64 years with ≥ 1 visit between 2012 and 2015 in 412 primary care CHCs in 9 expansion and 4 nonexpansion states. We assessed changes in rates of total, uninsured, Medicaid-insured, and privately insured primary care and preventive care visits; immunizations administered, and medications ordered. **Results:** Rates of uninsured visits decreased pre- to post-ACA, with greater drops in expansion (−57%) versus nonexpansion (−20%) states. Medicaid-insured visits increased 60% in expansion states while remaining unchanged in nonexpansion states. Privately insured visits were 2.7 times higher post-ACA in nonexpansion states with no increase in expansion states. Comparing 2015 with 2014: Uninsured visit rates continued to decrease in expansion (−28%) and nonexpansion states (−19%), Medicaid-insured rates did not significantly increase, and privately insured visits increased in nonexpansion states but did not change in expansion states. **Conclusions:** Medicaid expansion and subsidies to purchase private coverage likely increased the accessibility of health insurance for patients who had previously not been able to access coverage.

## Introduction

The Patient Protection and Affordable Care Act (ACA) was a landmark “natural policy experiment” in the United States, and there is controversy about the early outcomes of this experiment. Thus, it is crucial for scientists to study existing longitudinal datasets in order to better understand the impact of the ACA on insurance coverage rates and access to healthcare services. The ACA was developed to reach 47 million uninsured Americans and to make insurance more accessible and affordable for all Americans; the ACA created a new law mandating all US citizens and legal residents to have health insurance.^1^ Several provisions were put in place to assist with the health insurance mandate, including: (*a*) expanding Medicaid (public health insurance for low-income Americans) eligibility to cover adults earning up to 138% of the federal poverty level (FPL); (*b*) creating health insurance marketplaces where individuals can purchase private health insurance; and (*c*) providing credits and subsides for those purchasing private insurance through the marketplace for those making between 100% and 400% FPL.^2^ In 2012, the US Supreme Court made the Medicaid eligibility expansion optional for states. Consequently, 32 states including District of Columbia expanded their Medicaid programs as of July 2016 while 19 did not.^3^ Early studies revealed differences between expansion and nonexpansion states regarding insurance rates and healthcare utilization,^4-6^ but were limited by the inclusion of few states and had short follow-up time periods (6-12 months post-ACA).

This longitudinal, 4-year study makes a unique contribution to the literature by using electronic health record (EHR) data from community health centers (CHCs) in 13 states (9 expansion states; 4 nonexpansion states). CHCs (our nation’s health care safety net) provide health care by reducing barriers to cost, accepting uninsured patients, and vulnerable populations (eg, homeless, non-English speakers) serving nearly 25 million people yearly.^7^ Thus, CHCs are well suited to assess changes in insurance as they provide primary care to uninsured patients likely to gain access to coverage post-ACA.^8^ Additionally, research shows that primary care providers outside CHCs are not accepting or severely limiting acceptance of new Medicaid patients,^9-11^ while most CHCs are open to new Medicaid patients. Moreover, newly insured CHC patients are likely to continue receiving care from the CHCs.^12,13^ Furthermore, CHC EHR data include an objective measure of insurance status at visits, overcoming the limitation of recall bias or respondent’s understanding of the complex coverage system, commonly found in surveys.

This longitudinal study includes EHR data from a large population of CHC patients to assess changes in rates of uninsured, Medicaid-insured, and privately insured visits in expansion and nonexpansion states: 2 years pre- and 2 years post-ACA expansion. We hypothesized that rates of visits covered by insurance would be higher in the 2 years post-ACA versus the 2 years pre-ACA, and that this difference would be more pronounced in expansion states. We also suspected that patterns of visit coverage would continue to change in the second year post-ACA, relative to the first year post-ACA, and that patterns would be different in expansion versus nonexpansion states.

## Methods

EHR data were obtained from the Accelerating Data Value Across a National Community Health Center Network (ADVANCE) clinical data research network (CDRN) of PCORnet. The ADVANCE CDRN is a unique “community laboratory” for research with underrepresented populations receiving care in CHCs—our nation’s safety net. Led by the OCHIN (not an acronym) community health information network, the ADVANCE CDRN’s research-ready data warehouse integrates longitudinal outpatient EHR data from OCHIN, Health Choice Network (HCN), and Fenway Health, as described elsewhere.^14^ Data were collected on all nonpregnant patients (n = 875 571) aged 19 to 64 years with ≥1 ambulatory visit between 2012 and 2015 from 412 primary care CHCs “live” on their EHR system as of January 1, 2012 (n = 245 in 9 expansion states; n = 167 in 4 nonexpansion states). This analysis used ADVANCE CDRN data from OCHIN and HCN.

### Measures

The main independent variable was Medicaid expansion. As dates of Medicaid expansion were different for some states, we defined expansion states in our sample as those that expanded Medicaid on January 1, 2014 (including CA, HI, MD, NM, OH, OR, RI, WA, WI) and nonexpansion states as those who did not expand Medicaid through December 31, 2015 (including FL, KS, MO, NC). Although Wisconsin did not expand Medicaid to 138% FPL, it did open enrollment to adults with eligibility cireteria of 100% FPL acting more like an expansion than a nonexpansion state. Pre- and post-periods were defined as 2 years before, and 2 years after Medicaid expansion on January 1, 2014. To examine temporal changes following expansion, we further compared the first year (2014) to the second year (2015) post-expansion.

We included the following covariates associated with differences in health insurance status:^4,5^ sociodemographic variables (clinic-level distribtuions of sex, age, race/ethnicity, primary language, and federal poverty level) and state-level factors (marketplace type, 2013 minimum wage, 2013 uninsured rate, and 2013 unemployment rate).

### Outcome Measures

*Health care delivery* included rates of all billed encounters (all, primary care visits, new patient visits, and established patient visits) and receipt of preventive services (preventive medicine visits, immunizations, and medications ordered). Visit-type were determined using the primary Current Precedural Terminology (CPT) code for each visit. We assessed rates of uninsured, Medicaid-insured, and privately insured primary care visits.

### Analysis

Visit rates were computed by dividing the number of visits in a given interval by the total number of adult patients seen in a given clinic over the study period, scaled per 1000 patients per month. Post- versus pre-expansion rate ratios (RRs), and difference-in-difference (DD) ratios (comparing pre-post changes in rates between expansion groups) with 95% confidence intervals (CIs) were obtained from fitting generalized estimating equation (GEE) Poisson models with robust sandwich variance estimators for each outcome. GEE models included indicators for time and Medicaid expansion status, and an interaction term between these variables. We obtained linear combinations from the interaction term to estimate (*a*) post- versus pre-expansion rate ratios within expansion group, (*b*) difference-in-difference ratios (comparing post- versus pre-period changes between expansion states vs nonexpansion states), and (*c*) second year post- (2015) versus first year post-ACA (2014) rate ratios within expansion group. We clustered all models by CHC and used an autoregressive covariance structure to account for within-clinic temporal correlation. Models were adjusted for clinic-level demographic distributions and state-level factors. Analysis was conducted using SAS v.9.4 (SAS Institute, Inc); statistical significance was set at *P* < .05.

This study was approved by the Oregon Health & Science University Institutional Review Board.

## Results

Table 1 describes the patient population in the primary care clinics by expansion status. Overall, there was a greater proportion of female than male patients, more than 30% of the patient population was Hispanic, and the majority had incomes below 138% of the federal poverty level.

**Table 1.** Demographic Distribution in Community Health Centers in Nonexpansion and Expansion States, 2012-2015.

	Nonexpansion States^a^	Expansion States^b^
No. of community health centers	167	245
Total no. of patients	388 152	487 419
Total no. of primary care encounters	1 718 348	2 993 885
Patient demographics		
Gender, n (%)		
Female	240 223 (61.9)	271 306 (55.7)
Male	147 920 (38.1)	215 713 (44.3)
Other/unknown	9 (<0.1)	400 (0.1)
Age group (years), as of January 1, 2014, n (%)		
19-26	60 106 (15.5)	82 303 (16.9)
27-39	113 341 (29.2)	155 230 (31.9)
40-64	214 705 (55.3)	249 886 (51.3)
Race/ethnicity		
Hispanic	145 688 (37.5)	153 858 (31.6)
Non-Hispanic white	116 966 (30.1)	242 080 (49.7)
Non-Hispanic black	101 322 (26.1)	46 713 (9.6)
Other/unknown	24 176 (6.2)	44 768 (9.2)
Primary language, n (%)		
English	296 392 (76.4)	382 148 (78.4)
Spanish	84 710 (21.8)	86 100 (17.7)
Other/unknown	7050 (1.8)	19 171 (3.9)
Federal poverty level (last recorded), n (%)		
≤ 138%	304 612 (78.5)	323 604 (66.4)
>138%	45 970 (11.8)	73 814 (15.1)
Unknown	37 570 (9.7)	90 001 (18.5)

a Nonexpansion states: FL, KS, MO, NC.

b Expansion states: CA, HI, NM, OH, OR, RI, WA, WI.

**Figure 1.**
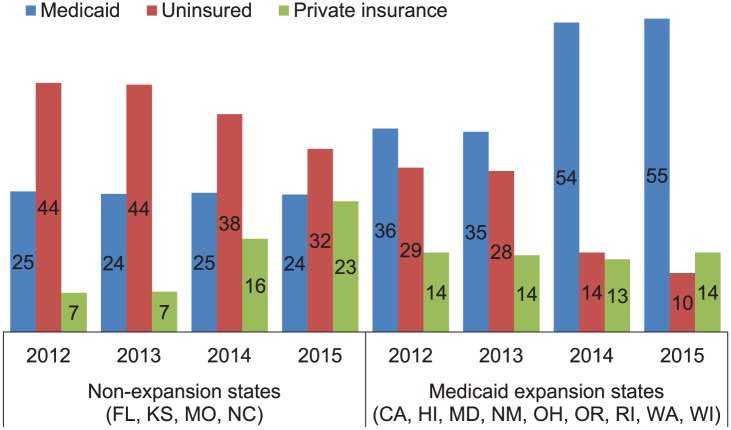
Distribution (%) of visit payer mix by expansion status and study year. Yearly totals do not sum to 100%; Medicare and other/miscellaneous coverage types comprise the remaining proportion of visits.

Figure 1 shows the distribution of visit payer mix by year and expansion status. The figure demonstrates a marked decrease in uninsured visits and and increase in Medicaid-insured visits in expansion states and privately insured visits in nonexpansion states.

### Two Years Pre- Versus 2 Years Post-ACA Medicaid Expansion

As seen in Table 2, rates of uninsured visits decreased from pre- to post-ACA by 57% (RR = 0.43, 95%CI = 0.38-0.49) in expansion versus 20% (RR = 0.80, 95% CI = 0.72-0.89) in nonexpansion states (DD = 0.54, 95% CI = 0.46-0.64; Table 2). Correspondingly, rates of Medicaid-insured visits increased 60% in expansion states while remaining unchanged in nonexpansion states (DD = 1.61, 95% CI = 1.43-1.82). Privately insured viits were 2.7 times higher post-ACA in nonexpansion states with no increase in expansion states (DD = 0.54, 95% CI = 0.46-0.64). Preventive care visits, immunizations administered, and medications ordered increased significantly in expansion states, but between-expansion group differences did not reach statistical significance.

**Table 2.** Community Health Center Utilization Rates and Rate Ratios Pre- and Post-ACA Medicaid expansions, in Expansion and Nonexpansion States.^a^

	24 Months Pre-ACA Rate (SE)	24 Months Post-ACA Rate (SE)	Post- vs Pre-ACA RR (95% CI)	DD Ratio (95% CI)	First 12 Months Post-ACA (2014) Rate (SE)	Second 12 Months Post-ACA (2015) Rate (SE)	2015 vs 2014 RR (95% CI)
Total primary care visits							
Nonexpansion	74.6 (7.2)	73.9 (7.5)	0.99 (0.92-1.07)		75.3 (7.5)	72.5 (7.5)	0.96 (0.91-1.02)
Expansion	78.4 (5.5)	81.6 (5.6)	1.04 (0.99-1.09)	1.05 (0.96-1.15)	83.4 (5.8)	79.9 (5.7)	0.96 (0.92-1.00)
Medicaid-insured visits							
Nonexpansion	20.4 (2.7)	20.2 (2.7)	0.99 (0.91-1.08)		20.8 (2.8)	19.7 (2.7)	0.95 (0.89-1.02)
Expansion	25.3 (2.8)	40.4 (3.8)	**1.60 (1.47-1.74)**	**1.61 (1.42-1.82)**	40.8 (3.8)	39.9 (3.9)	0.98 (0.94-1.02)
Uninsured visits							
Nonexpansion	28.3 (3.6)	22.6 (2.9)	**0.80 (0.72-0.89)**		25.1 (3.2)	20.4 (2.8)	**0.81 (0.76-0.87)**
Expansion	24.3 (2.7)	10.5 (1.4)	**0.43 (0.38-0.49)**	**0.54 (0.46-0.64)**	12.3 (1.9)	8.9 (1.0)	**0.72 (0.62-0.84)**
Privately insured visits							
Nonexpansion	5.8 (1.3)	15.8 (2.4)	**2.74 (2.07-3.63)**		13.5 (2.1)	18.6 (2.8)	**1.38 (1.21-1.57)**
Expansion	12.7 (1.4)	12.9 (1.4)	1.02 (0.90-1.15)	**0.37 (0.27-0.50)**	12.6 (1.4)	13.3 (1.5)	1.05 (0.99-1.12)
New patient visits							
Nonexpansion	6.8 (0.7)	6.3 (0.6)	0.92 (0.81-1.03)		6.8 (0.7)	5.8 (0.6)	**0.85 (0.78-0.93)**
Expansion	8.2 (0.5)	8.2 (0.5)	1.01 (0.91-1.11)	1.10 (0.94-1.28)	8.8 (0.5)	7.7 (0.6)	**0.88 (0.80-0.98)**
Established patient visits							
Nonexpansion	61.1 (6.9)	60.0 (7.1)	0.98 (0.91-1.06)		60.6 (7.1)	59.4 (7.2)	0.98 (0.93-1.03)
Expansion	74.4 (5.9)	77.7 (6.1)	1.04 (1.00-1.09)	1.06 (0.97-1.16)	78.6 (6.2)	76.8 (6.1)	0.98 (0.94-1.02)
Preventive medicine visits							
Nonexpansion	7.3 (1.5)	9.0 (1.8)	**1.23 (1.07-1.42)**		9.0 (1.9)	9.1 (1.8)	1.01 (0.90-1.14)
Expansion	3.2 (0.5)	3.7 (0.6)	**1.15 (1.06-1.25)**	0.93 (0.79-1.10)	3.7 (0.6)	3.7 (0.6)	0.99 (0.93-1.06)
Immunizations							
Nonexpansion	0.7 (0.1)	0.7 (0.1)	0.98 (0.87-1.10)		0.6 (0.1)	0.7 (0.1)	1.04 (0.96-1.13)
Expansion	1.8 (0.2)	2.0 (0.2)	**1.11 (1.03-1.20)**	1.14 (0.99-1.31)	1.9 (0.2)	2.1 (0.2)	**1.11 (1.02-1.20)**
Medications ordered							
Nonexpansion	23.4 (1.9)	24.1 (2.0)	1.03 (0.96-1.11)		24.6 (2.0)	23.6 (2.0)	0.96 (0.92-1.01)
Expansion	39.4 (1.9)	42.7 (2.1)	**1.09 (1.04-1.14)**	1.05 (0.97-1.15)	42.8 (2.1)	42.7 (2.1)	1.00 (0.97-1.03)

Abbreviations: RR, rate ratio; DD, difference-in-difference testing post- versus pre-period in expansion versus nonexpansion states; SE, standard error; ACA, Affordable Care Act.

a Nonexpansion states: FL, KS, MO, NC. Expansion states: CA, HI, MD, NM, OH, OR, RI, WA, WI. Visit rates calculated per 1000 patients per month; immunization and medication rates calculated per 1000 visits per month. Boldfaced values indicate statistically significant difference, *P* < .05. Primary care visits: CPT 99201-99205, 99212-99215, 99241-99245, 99385-99387, or 99395-99397 with MD, DO, NP, PA, midwife, or resident with no specialty listed. New patient visits: CPT 992201-99205. Established patient visits: CPT 99211-99215. Preventive medicine visits: CPT 99385-99387, 99395-99397. Immunizations: CPT 90460-90749. Medications: distinct RXNORM code per patient per day. Generalized estimating equation Possion models adjusted for clinic-level demographic distributions (sex, age, federal poverty level, primary language, race, and ethnicity) and state-level factors (marketplace type, 2013 minimum wage and unemployment rates, and 2013 uninsured rate), clustered by facility to account for within-facility correlation. RR and DD estimates obtained from linear combinations of time × expansion status interaction.

### First Year (2014) Versus Second Year (2015) Post-ACA Medicaid Expansion

Uninsured visit rates continued to decrease significantly in the second year post-ACA (2015) compared to the first year (2014) in both expansion and nonexpansion states (28% and 19% decrease, respectively). Privately insured visits increased significantly more in 2015 than in 2014 in nonexpansion states (RR = 1.38, 95% CI = 1.21-1.57) and remained unchanged in expansion states. Medicaid-insured visit rates did not significantly increase from 2014 to 2015. The rate of new patient visits in both expansion and nonexpansion states declined significantly in 2015 versus 2014. Immunizations increased 11% in 2015 over 2014 in expansion states, with no significant change in nonexpansion states.

## Discussion

This study confirms that uninsured CHC visit rates decreased in 2014-2015 (post-ACA) compared with 2012-2013 (pre-ACA). The decline was more pronounced in expansion states (59%), compared with nonexpansion states (20%). The corresponding increase in Medicaid-insured visits in expansion states suggest that the decline in uninsured visits was likely due to uninsured patients gaining Medicaid in these states. In nonexpansion states, the increase in privately insured visits likely contributed to fewer patients presenting without insurance.^4-6^ Of note, CHCs in nonexpansion states had a much larger percentage of overall visits from uninsured patients, as compared with CHCs in expansion states. This result suggests that relying on private insurance solely (rather than expanding Medicaid) does not eliminate access to care barriers for many vulnerable patients, and although uninsured patients in CHCs can receive care, they have fewer visits and are likely to forgo needed care.

It was projected that Medicaid enrollment and health care utilization for patients newly eligible for Medicaid would grow progressively over 3 years post-ACA,^15^ but our study and Medicaid enrollment data^16^ imply that it happened more quickly. Here, we found Medicaid-insured visit rates held constant from 2014 to 2015. This finding suggests that the influx of Medicaid beneficiaries in 2014 and the resulting increase in health care visits and expenditures may have stabilized (consistent with Medicaid enrollees reports)^16^ potentially due to efficient outreach and enrollment practices by CHCs. Outreach and enrollment is a crucial role of CHCs and with the implementation of ACA this role became even more essential. Many CHCs throughout the country received grants from the Health Resources and Services Administration to assist with outreach and enrollment efforts.^17^ Results from the present study suggest that these efforts were valuable and likely led to patients gaining Medicaid much earlier in the post-ACA period than predicted. Our finding that most patients sustained coverage into the second post-ACA year suggests these new policies had a positive impact on coverage retention as well.

Additionally, the preliminary cost of expanding Medicaid can now be measured more precisely, which is important to inform states that have (or have not) expanded their Medicaid programs and contribute useful information at this early stage to inform deliberations about the future of the ACA and other US health policy reforms.^18^

Privately insured visit rates increased substantially pre- to post-ACA in nonexpansion states and continued to rise through 2015. This finding demonstrates the importance of the ACA’s health insurance marketplaces in states that did not expand Medicaid. As 71% of CHC patients have incomes at or below the FPL, most were likely able to receive subsidies to purchase health insurance, making private coverage affordable. More research is needed to understand the affordability and acceptability of these private health insurance plans.

### Limitations

This study includes CHCs who are part of the ADVANCE CDRN; results may not be representative of all clinics, states, or expansion status groups. This visit-based analysis does not assess changes in patient-level insurance status or changes in patient panels. Our multivariable analysis adjusted to account for patient panel and economic differences, yet unmeasured confounders could affect our results such as clinic-specific insurance outreach efforts, private insurance details (eg, deductible), immigrant status, and patient panel health status (eg, comorbidity). Finally, the level of financial assistance (fees adjusted based on ability to pay) for uninsured patients varies by CHC and may explain the lack of change in uninsured visit rates in nonexpansion states.

## Conclusion

This study provides important information on the changing payer-specific utilization patterns in CHCs 2 years before through 2 years after implementation of ACA Medicaid expansions, comparing outcomes in expansion versus nonexpansion states. Findings from this study suggest that Medicaid expansion and subsidies to purchase private coverage likely increased the accessibility of health insurance for patients who had previously not been able to access coverage. Thus, the ACA was successful in its goal of increasing health insurance coverage, especially in states that expanded Medicaid. However, while CHC patients in expansion states benefited greatly from the Medicaid expansion, those in nonexpansion states only saw modest gains in coverage. These findings suggest that relying only on private insurance (rather than expanding Medicaid) is not a viable solution to provide sufficient coverage for vulnerable patients.
